# Supplementary figures and images for: A single dietary factor, daily consumption of a fermented beverage, can modulate the gut bacteria and fecal metabolites within the same ethnic community
Source: mSystems. 2023 Oct 26;8(6):e00745-23. doi: 10.1128/msystems.00745-23 (PMC10734539; doi:10.1128/msystems.00745-23)

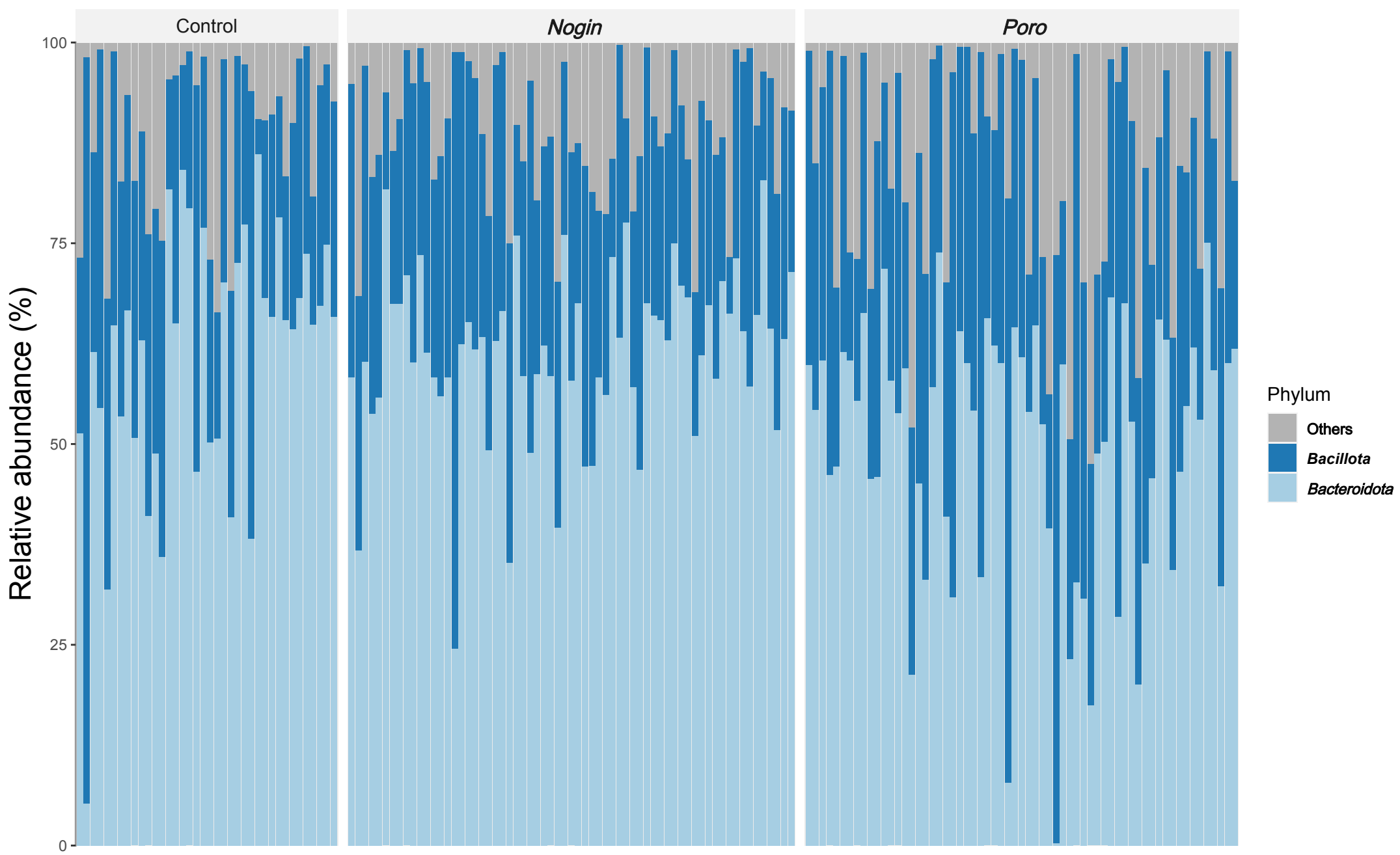

Supplement: Fig. S1 — Bacillota/Bacteroidota ratio. [file msystems.00745-23-s0001.pdf]

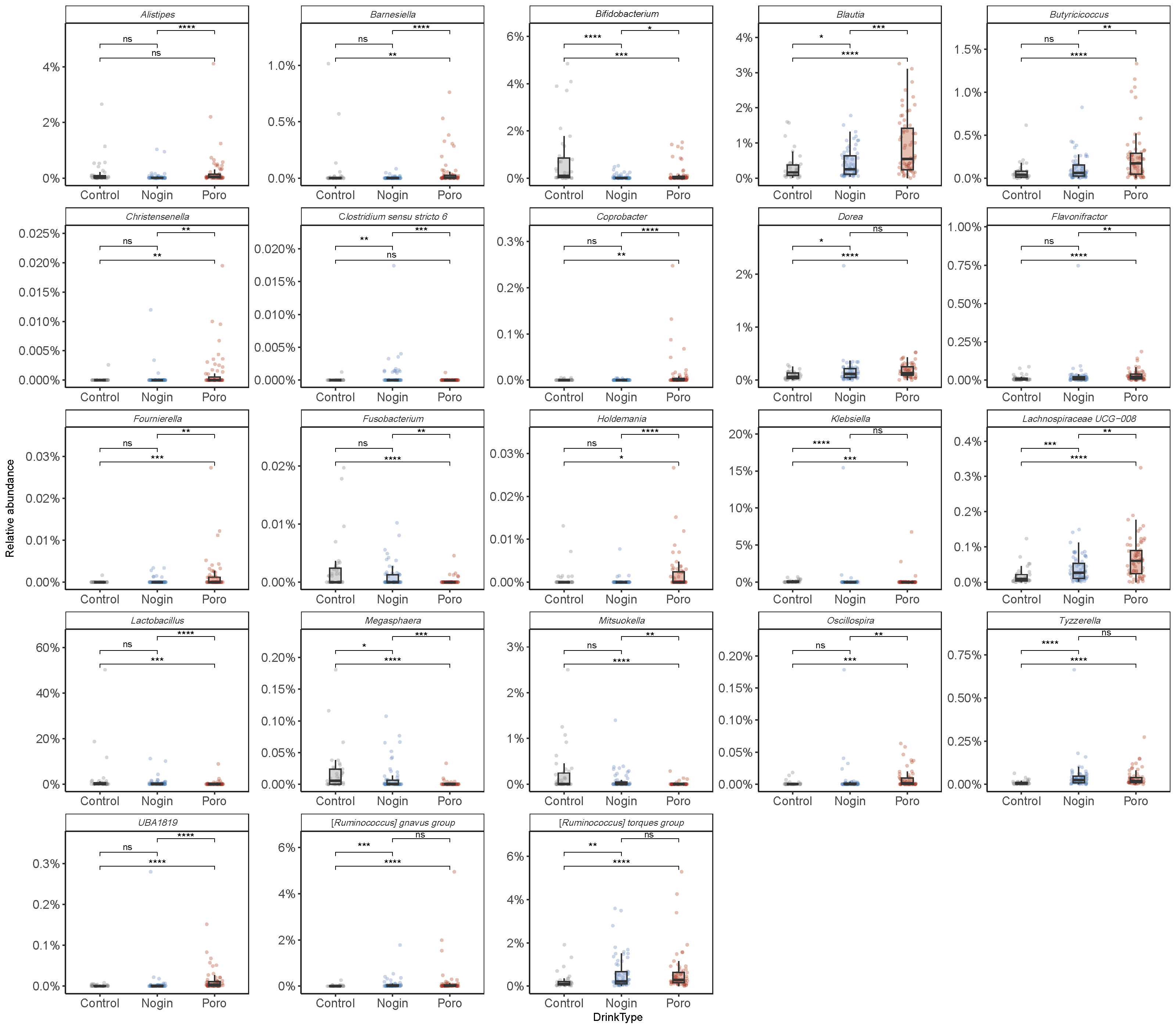

Supplement: Fig. S2 — Differentially abundant bacteria. [file msystems.00745-23-s0002.png]

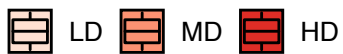

Ngin Drinkers

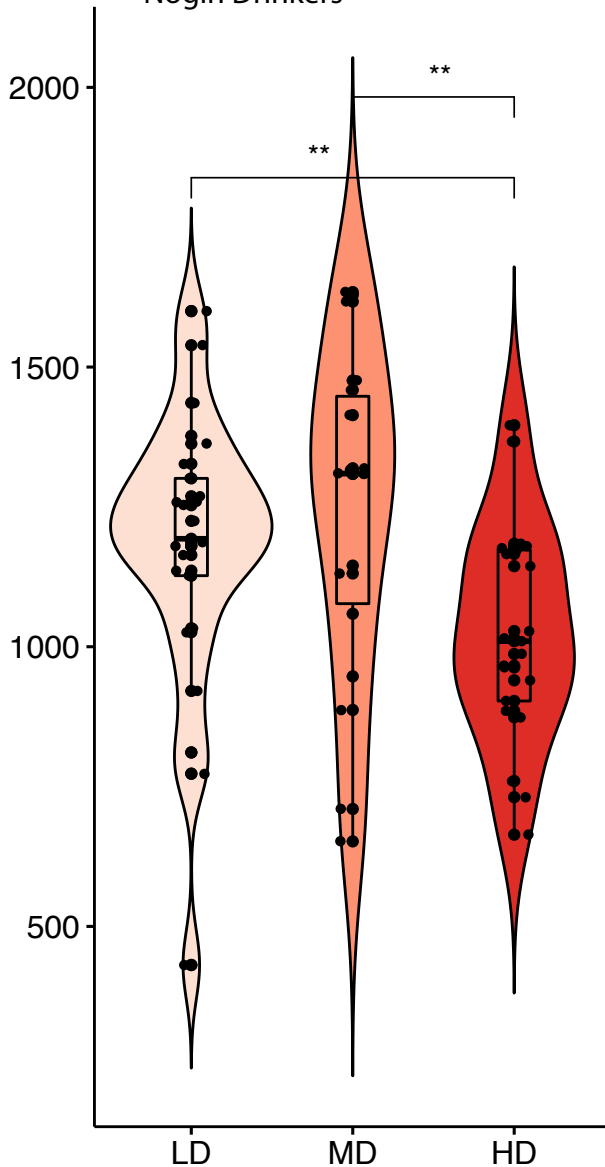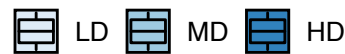

Poro Drinkers

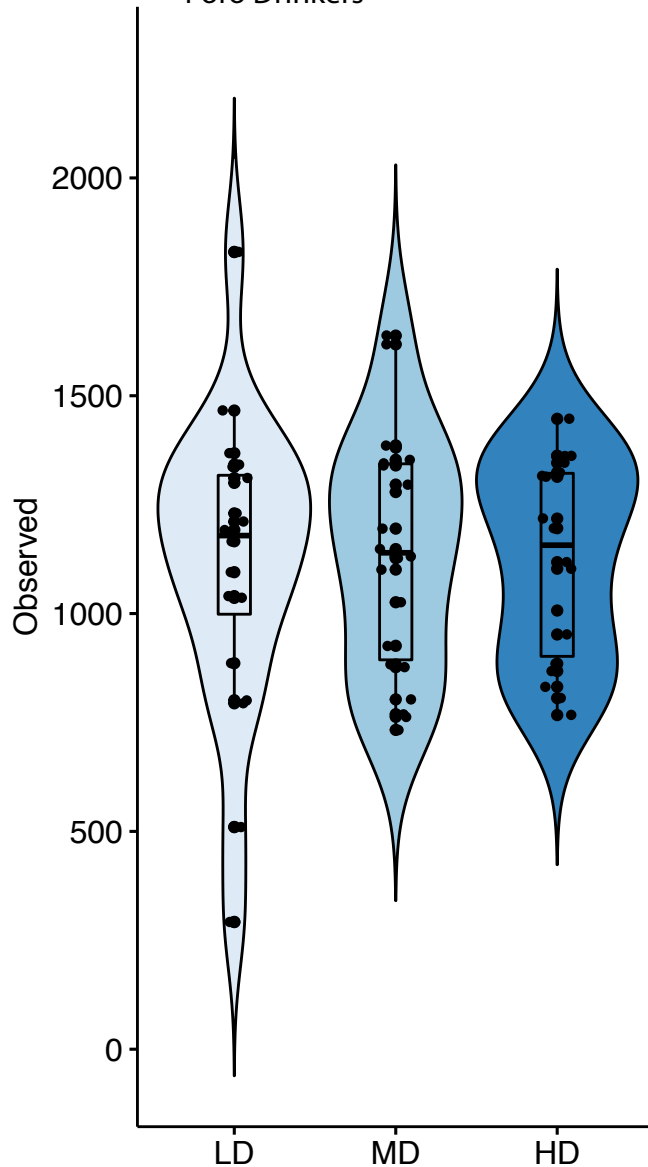

Supplement: Fig. S3 — Alpha diversity between LD, MD, and HD. [file msystems.00745-23-s0003.pdf]

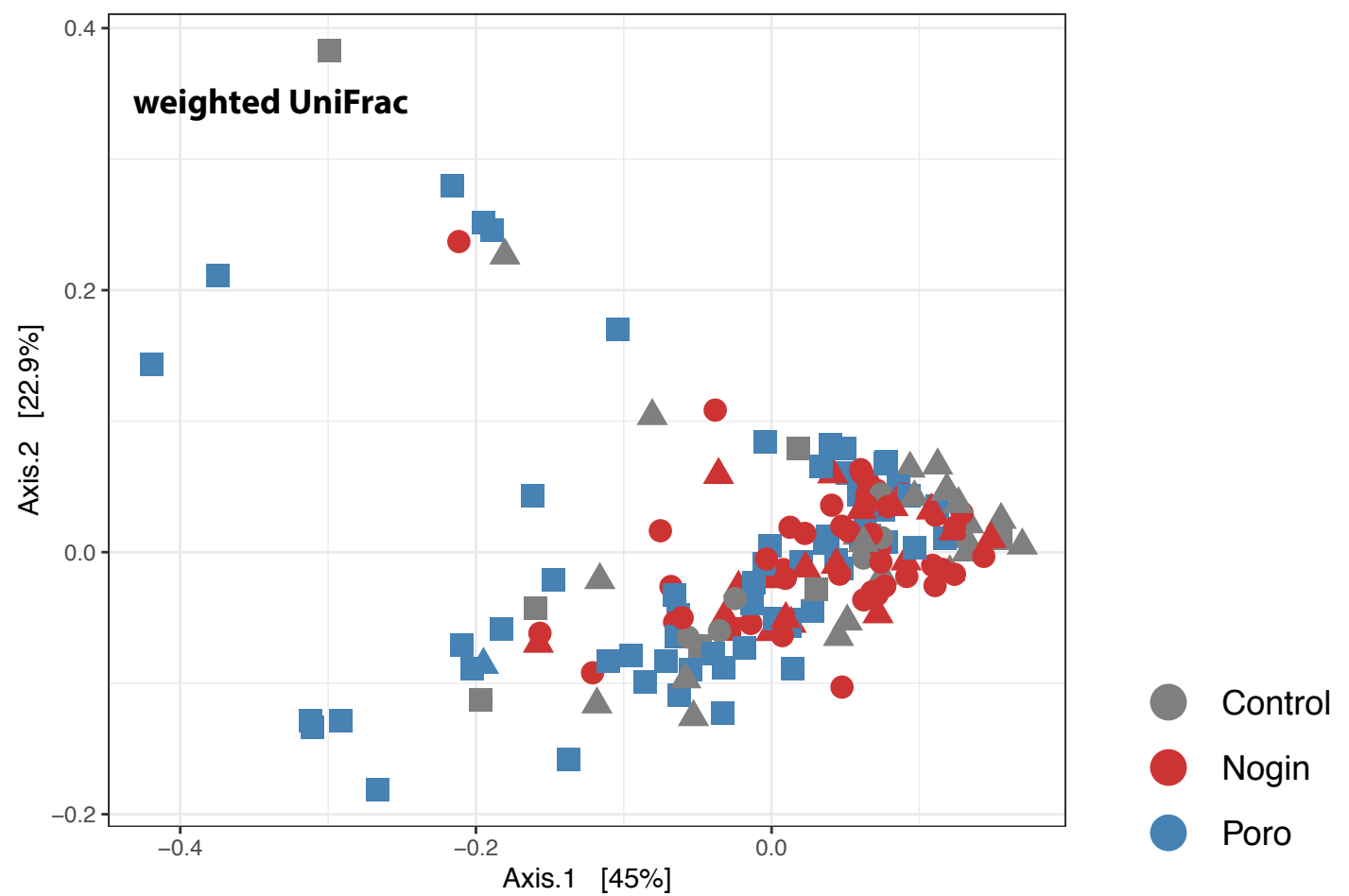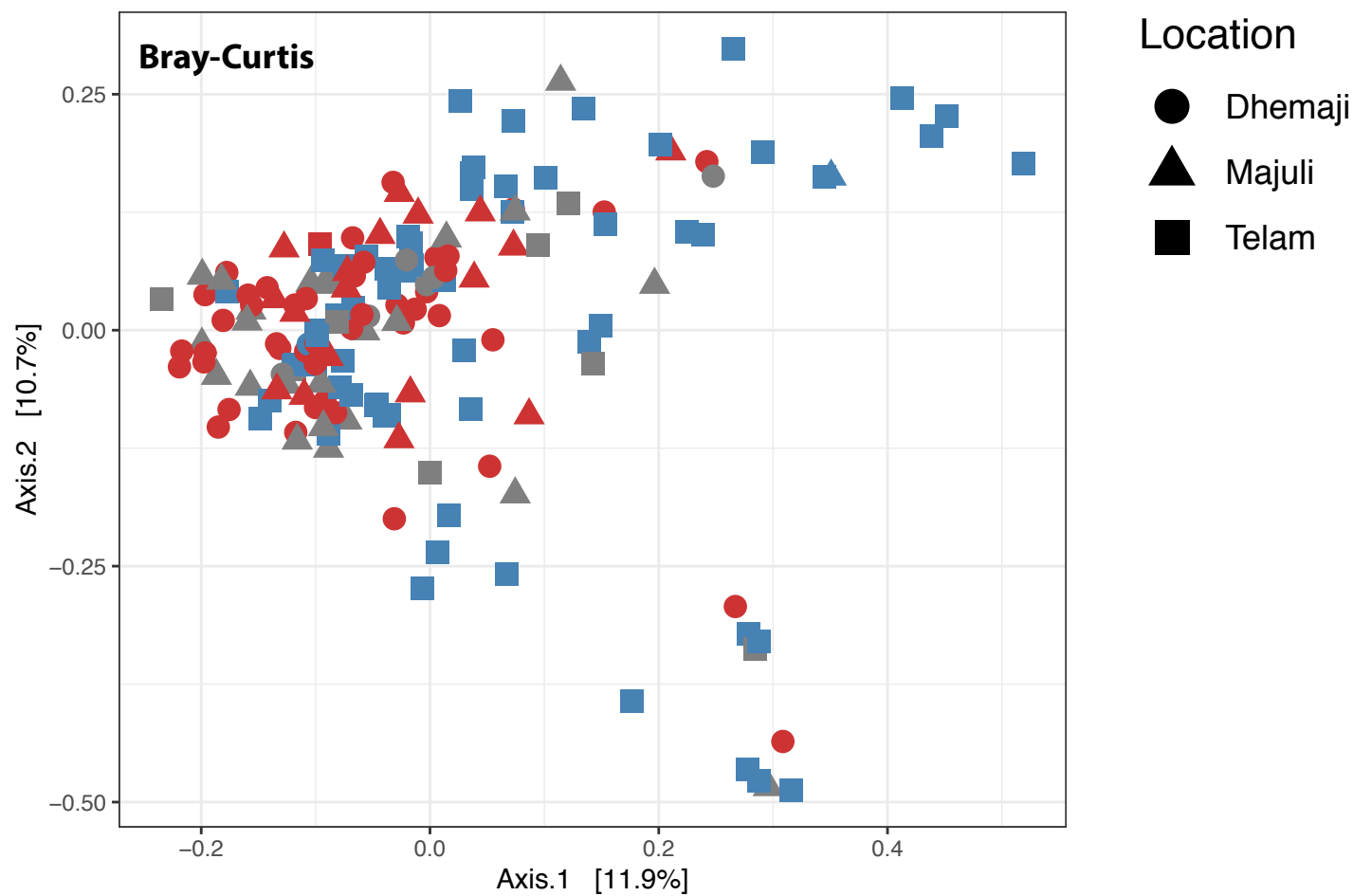

Supplement: Fig. S4 — PCoA of the weighted UniFrac and Bray-Curtis distances of the gut bacterial composition of Apong drinkers and non-drinkers. [file msystems.00745-23-s0004.pdf]

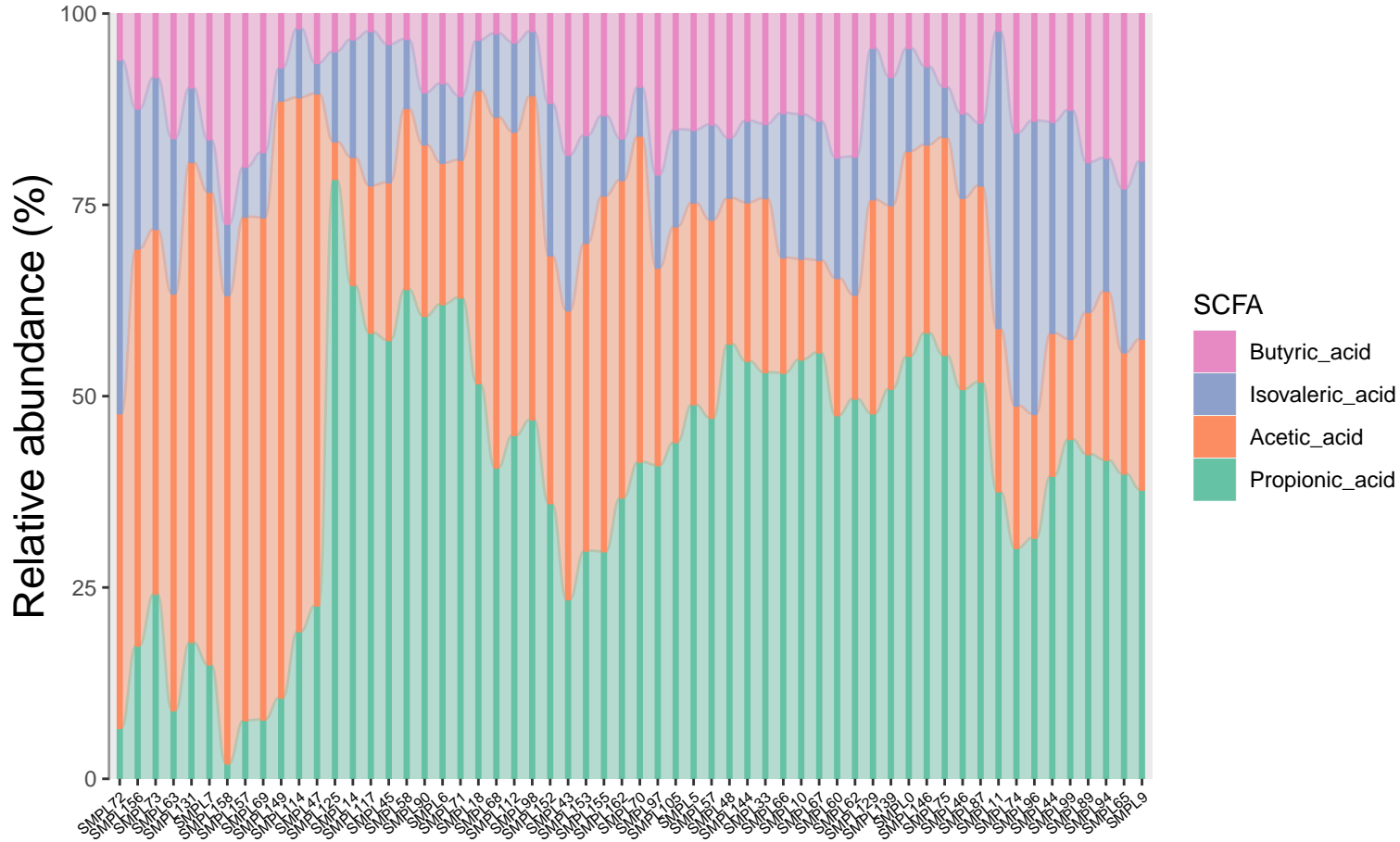

Supplement: Fig. S5 — Composition of the four short chain fatty acids (SCFAs). [file msystems.00745-23-s0005.pdf]
