# Supplementary material for: A single dietary factor, daily consumption of a fermented beverage, can modulate the gut bacteria and fecal metabolites within the same ethnic community
Source: mSystems. 2023 Oct 26;8(6):e00745-23. doi: 10.1128/msystems.00745-23 (PMC10734539; doi:10.1128/msystems.00745-23)
Supplement: Supplemental text — Legends for supplemental figures and tables. [file msystems.00745-23-s0006.docx]

**Supplementary Figure 1**: Stacked bar plot depicting the distribution of *Bacillota* and *Bacteroidota* across the samples.

**Supplementary Figure 2**: Differentially abundant bacterial taxa at the genus level among *Apong* drinkers and non-drinkers. The significant correlations are marked with *s, where *** depicts ≤ 0.001, ** depicts ≤ 0.01, and * depicts ≤ 0.05Only significant p-values are indicated.

**Supplementary Figure 3:** Microbial diversity of *Apong* drinkers estimated by ASV richness: “low-drinkers” (less than 250 ml per day), “medium-drinkers” (250-500 ml per day), and “high-drinkers” (more than 500 ml per day).

**Supplementary Figure 4:** PCoA of the weighted UniFrac and Bray-Curtis distances of the gut bacterial composition of *Apong* drinkers and non-drinkers where colors depict *Nogin*, *Poro*, and non-drinkers and shapes depict different locations.

**Supplementary Figure 5:** Composition of the four short chain fatty acids (SCFAs) in the fecal samples of the participants.

**Supplementary Table 1: Demographic information of the volunteers**

**Supplementary Table 2:** Correlations between gut microbial taxa at genus level and fecal metabolites.
